# Application of *in vitro* Drug Metabolism Studies in Chemical Structure Optimization for the Treatment of Fibrodysplasia Ossificans Progressiva (FOP)

**DOI:** 10.3389/fphar.2019.00234

**Published:** 2019-04-24

**Authors:** Elias C. Padilha, Jianyao Wang, Ed Kerns, Arthur Lee, Wenwei Huang, Jian-kang Jiang, John McKew, Abdul Mutlib, Rosangela G. Peccinini, Paul B. Yu, Philip Sanderson, Xin Xu

**Affiliations:** ^1^Division of Preclinical Innovation, National Center for Advancing Translational Sciences, National Institutes of Health, Rockville, MD, United States; ^2^Department of Natural Active Principles and Toxicology, School of Pharmaceutical Sciences, Universidade Estadual Paulista (UNESP), Araraquara, Brazil; ^3^Department of Pharmacokinetics, Dynamics and Metabolism, Discovery Sciences, Janssen Research and Development, Spring House, PA, United States; ^4^Frontage Laboratories, Inc., Department of Drug Metabolism, Exton, PA, United States; ^5^Division of Cardiovascular Medicine, Brigham and Women’s Hospital and Harvard Medical School, Boston, MA, United States

**Keywords:** metabolite identification, fibrodysplasia ossificans progressiva, structure optimization, aldehyde oxidase, reactive metabolite

## Abstract

Currently no approved treatment exists for fibrodysplasia ossificans progressiva (FOP) patients, and disease progression results in severe restriction of joint function and premature mortality. LDN-193189 has been demonstrated to be efficacious in a mouse FOP disease model after oral administration. To support species selection for drug safety evaluation and to guide structure optimization for back-up compounds, *in vitro* metabolism of LDN-193189 was investigated in liver microsome and cytosol fractions of mouse, rat, dog, rabbit, monkey and human. Metabolism studies included analysis of reactive intermediate formation using glutathione and potassium cyanide (KCN) and analysis of non-P450 mediated metabolites in cytosol fractions of various species. Metabolite profiles and metabolic soft spots of LDN-193189 were elucidated using LC/UV and mass spectral techniques. The *in vitro* metabolism of LDN-193189 was significantly dependent on aldehyde oxidase, with formation of the major NIH-Q55 metabolite. The piperazinyl moiety of LDN-193189 was liable to NADPH-dependent metabolism which generated reactive iminium intermediates, as confirmed through KCN trapping experiments, and aniline metabolites (M337 and M380), which brought up potential drug safety concerns. Subsequently, strategies were employed to avoid metabolic liabilities leading to the synthesis of Compounds **1**, **2,** and **3**. This study demonstrated the importance of metabolite identification for the discovery of novel and safe drug candidates for the treatment of FOP and helped medicinal chemists steer away from potential metabolic liabilities.

## Introduction

Fibrodysplasia ossificans progressiva (FOP) is a rare congenital disorder involving progressive and widespread postnatal ossification of soft tissues (Buyse et al., 1995). The heterotopic ossification occurs independently from normal bone and progresses in a well-defined spatial pattern. It forms ribbons, sheets and plates of bone that may fuse the joints of the individual. The ectopic calcification may also affect muscles, tendons and ligaments, causing severe pain, impairing mobility and typically leading to premature mortality. It affects approximately 1 in 2 million individuals (Shore and Kaplan, 2008). A congenital disorder, it usually manifests itself after physical trauma, surgery, viral illness or myositis during the first decade of life (Buyse et al., 1995; Yu et al., 2008a; Pignolo et al., 2013). Bearers hold no ethnic, racial, gender, or geographic predisposition (Pignolo et al., 2013). Rarely, genetic inheritance may be involved. When observed, genetic transmission is autosomal dominant and can be inherited from either mother or father.

The general cause of the disease is a heterozygous mutation in the ACVR1 gene which encodes the bone morphogenetic protein (BMP) type I receptor ALK2 (Shore et al., 2006; Yu et al., 2008a). Published studies indicate that greater than 97% of FOP patients carry a specific ALK2 R206H mutation. This mutation disrupts an α-helix in the glycine-serine regulatory domain, rendering ALK2 constitutively active (Shore et al., 2006; Groppe et al., 2007; Yu et al., 2008a). When activated, BMP type I receptors (e.g., ALK2, ALK3, and ALK6) phosphorylate BMP-responsive SMADs (1/5/8) which signal the transcription of genes related to cell growth and differentiation to form bone. Recent studies found that the inflammatory stimulus of activin A is necessary to trigger the heterotopic bone formation in FOP. Normally, activin A has inhibitory activity on ALK2 mediated signaling, but it was found to have excitatory activity in ALK2^R206H^ patients, triggering heterotopic ossification (Hatsell et al., 2015; Hino et al., 2015; Alessi Wolken et al., 2018). Currently no effective drug treatment has been approved for FOP.

**FIGURE 1:**
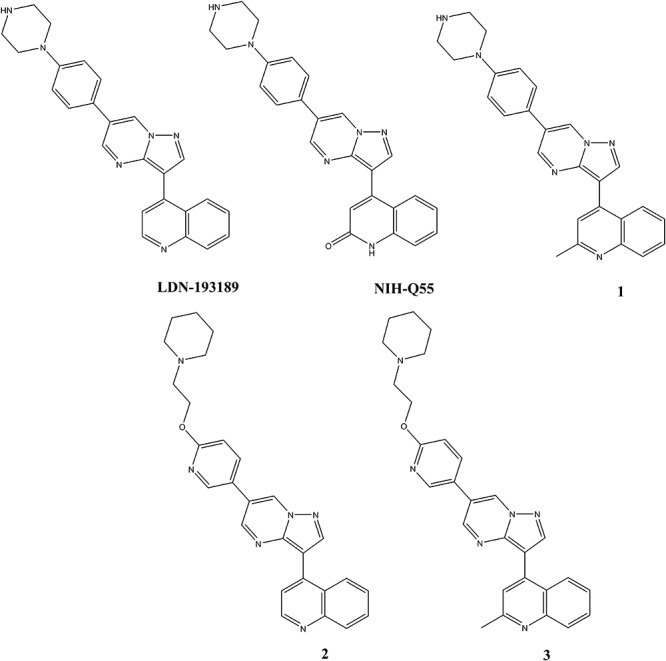
Chemical structures of test articles.

Addressing BMP receptor mediated processes, Yu and coworkers screened over 7,500 compounds and found that dorsomorphin perturbs dorsoventral axis formation in zebrafish (Yu et al., 2008b). The compound was able to inhibit SMAD dependent responses by inhibiting BMP receptor type I over-activation. Cuny and coworkers modified dorsomorphin to an optimized molecule LDN-193189 (Figure 1) which had moderate pharmacokinetic characteristics following intraperitoneal administration in mice (e.g., plasma t_1/2_ = 1.6 h) (Cuny et al., 2008). Later, this compound was shown to be efficacious in an animal model of FOP (Yu et al., 2008a).

In drug discovery, around 50% of the major causes of failure are due to poor pharmacokinetics and toxicity (van de Waterbeemd and Gifford, 2003). Optimization of a lead compound to make it a drug candidate involves many structure design and synthesis cycles that include pharmacological, pharmacokinetic and toxicological analyses, with the objective of identifying a compound with satisfactory characteristics for the entrance into drug development stage (Gunaratna, 2001; Hyland et al., 2006). Metabolite identification (MetID) sheds light on the biotransformation of a lead compound, sources of metabolic instability, the generation of potentially toxic metabolites, and where to propose modifications of the structure to improve upon the undesired characteristics. Therefore, MetID is a powerful tool for lead optimization (Gunaratna, 2001; Zhang et al., 2009; Zhao et al., 2017).

Although Cytochrome P450 (CYP) mediated pathways are the main focus during MetID studies, non-CYP mediated pathways are of growing importance. One major example is aldehyde oxidase (AO), which is responsible for the bioconversion of many xenobiotics (Pryde et al., 2010). AO is a molybdoflavoprotein, structurally related to the molybdenum cofactor (MoCo) containing family which also includes xanthine oxidase (XO), another known non-CYP enzyme. They are cytosolic enzymes mainly found in the hepatocytes of many mammals (Hutzler et al., 2013). As its name suggests, AO is involved in the oxidation of aldehydes to carboxylic acids. However, it is also involved in the oxidation of nitrogen-containing heterocyclic compounds, and in the reduction of nitro-aromatic compounds and isoxazole and isothiazole ring systems (Garattini and Terao, 2012; Romao et al., 2017). An increasing number of newly synthesized compounds are found to have some extent of metabolism by AO to the point that metabolism studies should not ignore this enzyme during drug discovery (Obach et al., 2004; Garattini and Terao, 2011; Zetterberg et al., 2016). Since FOP lead compound LDN-193189 has multiple sites potentially susceptible to AO oxidation, it was necessary to assess the metabolic impact of this enzyme during the structure optimization process.

To minimize the potential liability related to drug safety, it is important to assess the formation of reactive metabolites in discovery MetID studies. Reactive metabolites are electrophilic intermediates or radicals which are not easily identified without the proper methodology (Ma and Subramanian, 2006; Rousu et al., 2009). Unidentified reactive metabolites could be responsible for many serious, unpredicted drug reactions that have in the past led to the withdrawal of several drugs from the market (Kassahun et al., 2001; Olsen et al., 2005; Alvarez-Sánchez et al., 2006). The difficulty with identifying reactive metabolites is that they are unstable, usually forming in small amounts and reacting rapidly with nucleophiles. They may be hard electrophiles that react with hard nucleophiles such as basic groups in DNA and lysine residues in proteins, or soft electrophiles that react with soft nucleophiles such as thiol groups of glutathione and cysteine residues in proteins. In either case, their reaction with endogenous components may lead to loss of protein activity or to the generation of a compound-protein hapten which the immune system will react against, damaging cells or tissues (Ju and Uetrecht, 2002; Kalgutkar et al., 2008). The addition of glutathione to the MetID experiment is an effective mean to “trap” soft electrophilic reactive metabolites as relatively stable adducts for identification by LC-MS. The addition of KCN to the metabolism experiment will “trap” hard electrophiles for further characterization (Ma and Subramanian, 2006; Rousu et al., 2009).

The purpose of this study was to identify the metabolites of lead compound LDN-193189 through the incubation with liver microsomal and cytosolic fractions from mouse, rat, dog, rabbit, monkey and human. Information gained from the MetID study will be used to guide the medicinal chemistry efforts to generate new compounds with the liabilities identified in the MetID studies mitigated.

## Materials and Methods

### Synthesis of Compounds

LDN-193189 hydrochloride, NIH-Q55 (M422c), Compounds **1**, **2,** and **3** were provided by the medicinal chemistry team of NCATS/NIH with purity >98%. The structures for these five compounds are shown in Figure 1.

### Reagents

Pooled liver microsomes and cytosolic fractions from different species were obtained from Xenotech (Kansas City, KS), including CD-1 mice (Lot# 0310190 and Lot# 0610024), rats (Lot# 0710104 and Lot# 0710103), dogs (Lot# 452601 and Lot# 0710436), rabbits (Lot# 0810371 and Lot# 0510023), monkeys (Lot# 0510003 and Lot# 0310071), and humans (Lot# 452161 and Lot# 01610370), respectively. The following reagents obtained from Sigma Aldrich (St. Louis, MO, United States) were used for *in vitro* studies: MgCl_2_, NADPH-regenerating systems (NADP^+^, glucose-6-phosphate, and glucose-6-phosphate dehydrogenase), 0.1 M phosphate buffer (pH 7.4), GSH (glutathione, reduced), KCN, menadione (aldehyde oxidase inhibitor), allopurinol (xanthine oxidase inhibitor) and UDPGA. Solvents used for chromatographic analysis were HPLC or ACS reagent grade and purchased from EMD Chemicals (Gibbstown, NJ, United States) or other commercial suppliers. All other reagents were analytical or ACS reagent grade.

### Preparation of Stock Solutions

The stock solution (5 mM) of LDN-193189 was made by adding 0.877 mL of DMSO/acetonitrile (ACN; 50/50, v/v) into a vial containing 1.78 mg of the compound. Further dilution was made by using 100% ACN to make a 1 mM solution which was used for the incubations. Stock solutions for Compounds **1**, **2**, and **3** were prepared in a similar way.

### Incubations With Liver Microsomes and Cytosol From Different Species

LDN-193189 (10 μM) was incubated with mouse, rat, dog, rabbit, monkey and human liver microsomes (1 mg/mL) with/without cytosol (2 mg/mL) in the presence of an NADPH-regenerating system [(glucose-6-phosphate (3.6 mM), NADP^+^ (1.3 mM), and glucose-6-phosphate dehydrogenase (0.4 units/mL)], MgCl_2_ (10 mM), and UDPGA (2 mM) in 0.1 M phosphate buffer (pH 7.4), a procedure similar to previously described MetID workflows (Li et al., 2016; Ahire et al., 2017). Liver microsomal or cytosolic fractions containing appropriate cofactors were also fortified with either KCN (0.1 mM), GSH (2 mM), menadione (1 mM) or allopurinol (0.1 mM). Total incubation volume per sample was 1 mL. The metabolic reactions were initiated by the addition of cofactors, NADPH-regenerating system and UDPGA, after a pre-incubation at 37°C for 5 min. The incubation mixtures were placed in a shaking water bath at 37°C for 60 min. At the end of the reaction, three volumes of ACN were added, followed by vortexing and centrifugation to remove proteins. The supernatants were transferred to clean tubes and dried completely under a stream of nitrogen at ambient temperature. The dried residues were reconstituted with 500 μL of 25% MeOH in water and transferred into HPLC vials for LC/UV/MS analysis.

The Compounds **1**, **2,** and **3** were tested under the same conditions described for LDN-193189. However, only mouse and human *in vitro* preparations were used to determine whether these new compounds would improve the metabolic liabilities identified for LDN-193189. Control incubations in the absence of co-factors were conducted under similar conditions. Testosterone (100 μM) was incubated with human liver microsomes as a positive control to demonstrate the viability of the microsomal preparations and the incubation conditions used for the test compounds. However, the incubation time was reduced to 20 min. Acetaminophen (10 μM) and nicotine (10 μM) were used as positive controls for GSH and KCN trapping studies, respectively.

### LC/UV/MS Conditions for Metabolite Profiling and Identification

Metabolic profiling and characterization of LDN-193189 and Compounds **1**, **2,** and **3** in microsomal incubation extracts were performed using an LC/MS system consisting of a Surveyor HPLC system equipped with an autosampler and a diode array detector interfaced to an LTQ ion trap mass spectrometer (Thermo Fisher Scientific, San Jose, CA, United States). Chromatography was accomplished on a Phenomenex Luna, C18 (2) column, 3.0 × 250 mm, 5 μm (Torrance, CA, United States). The column was kept at ambient temperature during sample analysis. The mobile phases were HPLC grade water (solvent A) and ACN (solvent B), both of which contained 0.1% trifluoroacetic acid (TFA). A 1 h gradient from 5 to 95% of solvent B was applied at a flow rate of 0.3 mL/min. The first 4 min of the HPLC flow was diverted to waste prior to evaluation of metabolites.

To facilitate distinction of carbon or heteroatom oxidations, hydrogen/deuterium (H/D) exchange experiments were performed by replacing the aqueous mobile phase (H_2_O) with D_2_O while keeping the rest of assay conditions unchanged.

UV absorption spectra from 200 to 400 nm were recorded using a diode array detector. The Thermo Finnigan LTQ XL mass spectrometer was equipped with an electrospray ionization (ESI) interface and operated in positive ionization mode for metabolite profiling and identification. Mass spectra were acquired in full scan (MS) (m/z 200–1000) and data dependent scan (MS^2^, MS^3^, and MS^4^) modes. The ESI spray voltage was +5kV with capillary temperature of 300°C. Sheath gas and auxiliary gas were 80 and 20 arbitrary units, respectively. Activation Q was 0.25 and Activation time 30 ms. The collision energy used to obtain fragments was set at 40 eV.

### *In vitro* ADME and Activity Assays

*In vitro* metabolic stability screening assay was conducted with rat liver microsomes in a 96-well plate, where 1 μM of compound was incubated with 0.5 mg/mL of rat liver microsomal protein. A single time point was collected at 15 min after an incubation at 37°C. The percent parent drug remaining at 15 min was analyzed by LC/MS and half-life (t_1/2_) was calculated. When the half-life was greater than 30 min, results were presented as >30 min.

Kinetic solubility was measured using the μSOL Evolution system from pION Inc. (Billerica, MA, United States). Saturation shake-flask solubility method was adapted as previously described (Avdeef and Berger, 2001). Briefly, 10 mM DMSO stock was diluted to a final drug concentration of 150 μM in the aqueous solution (pH 7.4, 100 mM phosphate buffer) in duplicates. The solution was kept at room temperature for 6 h for equilibration. After equilibration, precipitates were removed using Tecan Te-Vac vacuum-filter and the compound concentration was measured by UV absorbance (λ: 250–498 nm). Data was compared to a fully solubilized reference solution of the test article at 17 μM in spectroscopically pure *n*-propanol. The kinetic solubility (μg/mL) was calculated using the μSOL Evolution software.

Parallel Artificial Membrane Permeability Assay (PAMPA) was employed to determine the passive diffusion permeability of LDN-193189 and analogs. Stirring double-sink PAMPA method (pION Inc.) was employed to determine the permeability of compounds. The method was performed as described previously (Sun et al., 2017).

The *in vitro* activity toward to ALK2 and the selectivity over ALK3 and ALK5 were determined in C2C12 cell lines (ATCC; Manassas, VA, United States) through measuring the phosphorylation level of SMAD1/5/8 (for ALK2 and ALK3) or SMAD2/3 (for ALK5) under the stimulation of BMP6 (mediated by ALK2), BMP4 (mediated by ALK3) and TGFβ (mediated by ALK5), respectively, by using homogeneous time resolved fluorescence (HTRF; a manuscript in preparation).

### Data Analysis

Xcalibur (version 2.07) was used to acquire and process mass spectral and UV absorption data.

## Results

### Metabolite Identification of LDN-193189 in Different Species

**FIGURE 2:**
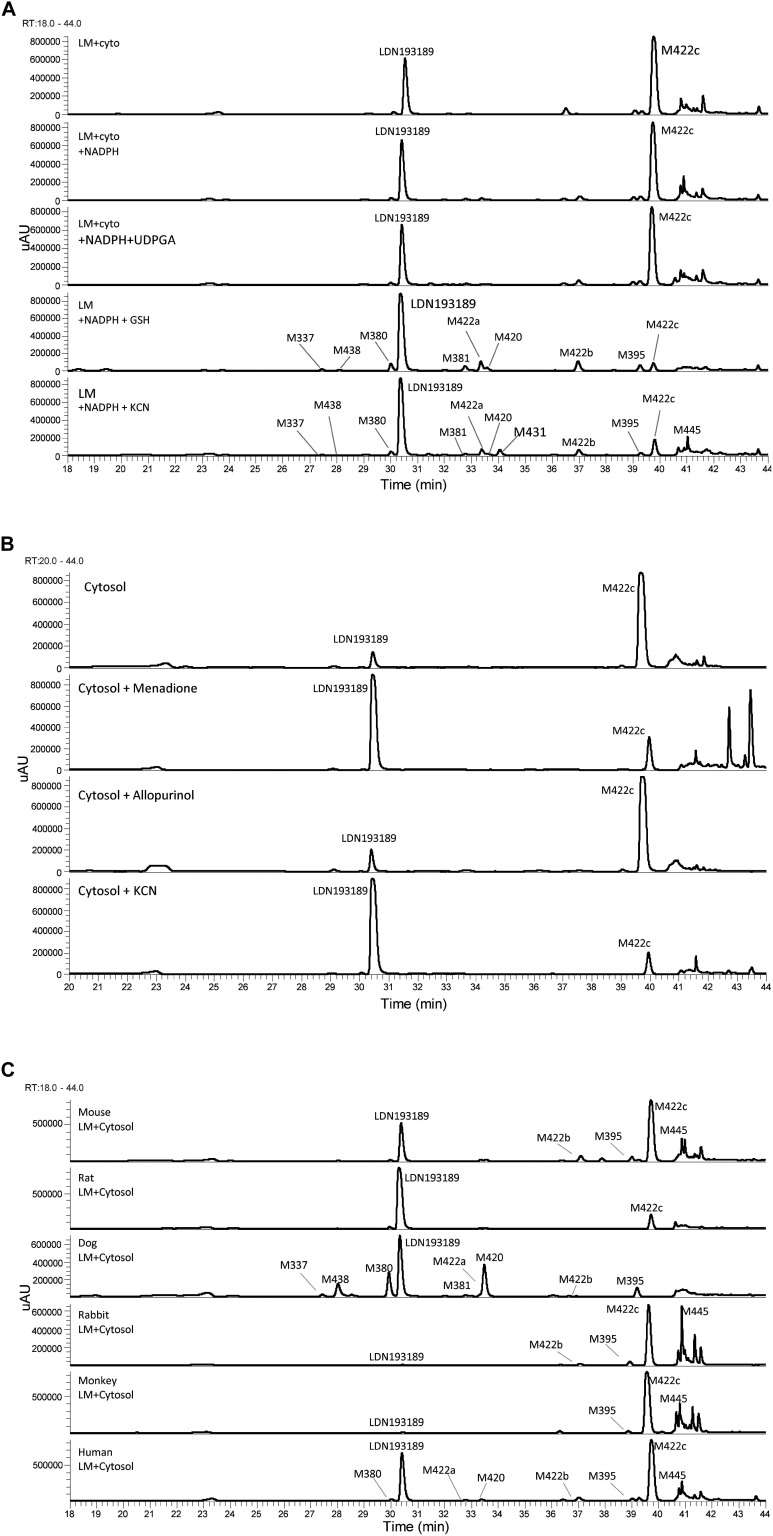
Metabolic profiles (LC/UV) of LDN-193189. **(A)** Incubations with human liver microsomal and cytosolic fractions in the presence of NADPH, UDPGA, GSH, and KCN (“LM”: Liver microsomes; “cyto”: cytosol). **(B)** Inhibition of the formation of M422c by menadione in human liver cytosol. **(C)** LDN-193189 incubation with liver microsomal and cytosolic fractions from mouse, rat, dog, rabbit, monkey, and human.

**FIGURE 3:**
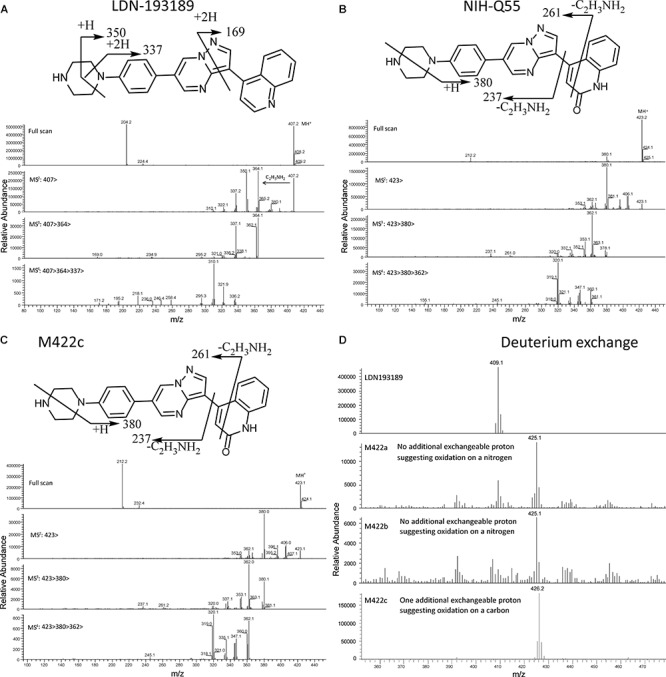
**(A)** Mass spectrum and fragment analysis of LDN-193189. **(B)** Fragment analysis and MS^4^ spectrum of the synthetic standard of the proposed AO-mediated metabolite, NIH-Q55. **(C)** MS^4^ spectrum and fragment analysis of the AO-mediated metabolite, M422c, with similar fragmentation patterns compared to NIH-Q55. **(D)** Deuterium exchange experiment showing that oxidation of M422a and M422b are nitrogen oriented while M422c is carbon oriented.

The metabolic profiles of LDN-193189 were obtained following incubations with mouse, rat, dog, rabbit, monkey and human liver microsomal/cytosolic preparations using LC/UV/MS analysis. Metabolic profile (LC/UV, with λ 310–315 nm) of LDN-193189 obtained for human is shown in Figure 2A. The identities of the metabolites displayed in the chromatograms were obtained by fragment analysis of the MS^n^ spectrum of each peak as shown for parent in Figure 3A. For metabolite fragment ions and rationale refer to Supplementary Table 1. The major metabolite observed in the UV chromatogram was M422c. The formation of M422c appeared to be mediated via a cytosolic enzyme through oxidation of the α-carbon of the quinoline, suggesting that it was produced by either aldehyde oxidase (AO) or xanthine oxidase (XO). The identity of M422c was unequivocally established by comparison with synthetic standard NIH-Q55 (see Figure 3B,C). Furthermore, formation of M422c in the presence of human liver cytosol was reduced by AO inhibitor menadione, confirming the involvement of AO. On the other hand, the addition of the XO inhibitor allopurinol did not result in a change in the formation of M422c (see Figure 2B), indicating no involvement of XO on the metabolism of LDN-193189. As shown in the Figure 2C the cytosolic fractions of all species except dog formed the major M422c metabolite.

**Table 1:** Summary of metabolites of LDN-193189.

Parent compound/Metabolites	RT (min)^a^	MH^+^	Biotransformation pathways	Species
LDN-193189	30.4	407	–	M, R, Rb, D, Mo, H
M337	27.5	338	*N,N*-dealkylation	M, Rb, D, Mo, H
M438	28.0	439	Oxidation	M, R, Rb, D, Mo, H
M380	30.0	381	*N,N*-dealkylation	M, R, Rb, D, Mo, H
M381	32.8	382	*N,N*-dealkylation	Rb, D, Mo, H
M422a	33.4	423	*N*-oxidation	M, R, Rb, D, Mo, H
M420	33.6	421	Oxidation	M, R, Rb, D, Mo, H
M431	34.1	432	Cyanide adduct of reactive intermediate	M, R, Rb, D, Mo, H
M422b	37.0	423	N-oxidation	M, Rb, D, Mo, H
M395	39.3	396	N,N-dealkylation	Rb, D, Mo, H
M422c	39.8	423	Oxidation	M, Rb, Mo, H
M445	41.0	446	Oxidation of M431	M, R, Rb, D, Mo, H

*Following Incubations in Mouse, Rat, Rabbit, Dog, Monkey and Human Liver Microsomal and Cytosolic Fractions. ^a^Retention time (RT) based on LC/MS data of HLM+NADPH+KCN. M, Mouse; R, Rat; Rb, Rabbit; D, Dog; Mo, Monkey; H, Human.*

**FIGURE 4:**
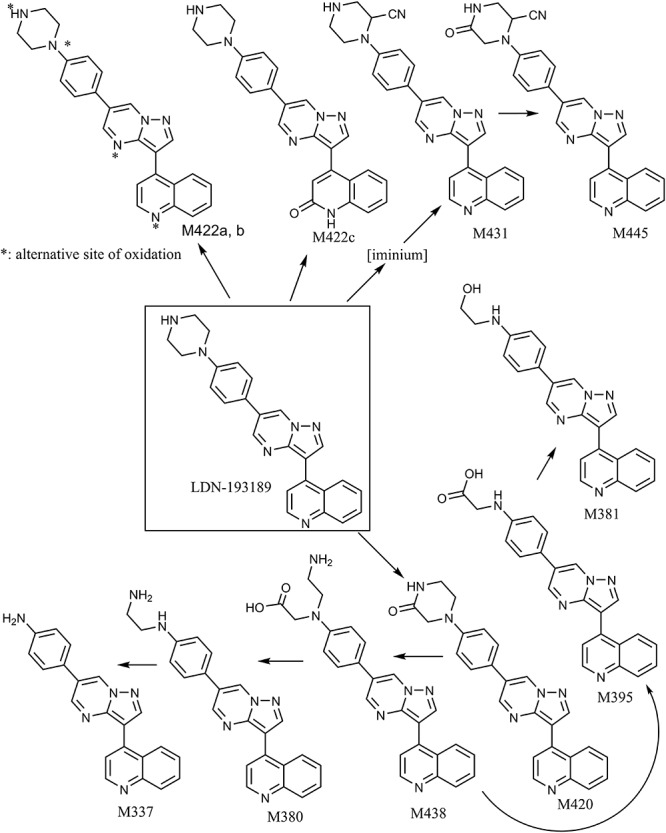
Proposed metabolic pathways of LDN-193189 after incubation with liver microsomes and cytosol from mice, rats, rabbits, dogs, monkeys and humans. M337 is an aniline derivative that conceals potential toxicity. M422c is formed via aldehyde oxidase metabolism and is the major metabolite. M445 and M431 are reactive metabolites trapped by cyanide.

In general, LDN-193189 was also found to be metabolized fairly extensively in some species even when cytosol was omitted from the microsomal incubations. The major metabolic pathway mediated by microsomal enzymes appeared to be via modification of the piperazine moiety. For example, in dog liver microsomes M420 and M380 appeared to be the major metabolites, produced as a result of piperazine ring oxidation followed by sequential dealkylation. Di-dealkylation of the piperazinyl ring led to the formation of aniline M337. Both M337 and M380 were not found in rat microsomal incubation with LDN-193189. Interestingly, the metabolite profiles obtained in this study showed that the compound exhibited greatest metabolic stability in the presence of rat liver microsomes (with and without cytosol). The metabolic pathway of LDN-193189 is depicted in Figure 4.

The MS^n^ spectrum of major metabolite peaks found in the LC/UV overview was obtained and the fragment analysis was performed in order to obtain the approximate structure of each metabolite. The structure of the major metabolite, M422c, was proposed by fragment analysis of the cytosolic incubation chromatogram peak with 39.8 min retention time (Figure 3C). Later, we used a deuterium exchange experiment to assess the characteristics of each mono-oxidized metabolite. Figure 3D shows that LDN-193189 has one exchangeable hydrogen at the piperazine moiety. An oxidation on carbon would add an exchangeable hydrogen. However, M422a and M422b also present one exchangeable hydrogen, which means that the oxidations occurred at nitrogen. Their MS^n^ spectra showed that the oxidation occurred at either nitrogen of the piperazinyl group. In contrast to M422a and M422b, the AO-metabolite M422c presents two exchangeable hydrogens, indicating that the hydroxylation occurred on a carbon. Its MS^n^ spectrum shows that the carbon is in the quinoline moiety, and the α-quinoline carbon was confirmed by comparison with NIH-Q55. The NIH-Q55 fragment analysis showed a similar profile to M422c (Figure 3B) and the retention times matched.

Following incubations with liver microsomes, LDN-193189 was mainly metabolized on the piperazine moiety, producing various piperazine ring-cleaved derivatives including M337, M380, M381, M395, and M438. Interestingly, there were no glucuronide conjugates observed in this study despite the formation of hydroxylated metabolites. Cyanide conjugate M431 and a further oxidized derivative M445 were observed in all species when KCN was added to the incubation mixtures. The fragmentation pattern of M431 suggests that the bioactivation took place on the piperazine moiety of the molecule (see Supplementary Table 1). Trapping studies conducted with GSH showed that no “soft electrophiles” were produced from LDN-193189 in the presence of liver microsomes from any species.

The proposed metabolic pathways of LDN-193189 are presented in Figure 4. All metabolites observed in human liver preparations were also produced in other non-clinical species, a relevant information for the species selection and interpretation of pre-clinical toxicity study results. A list of the metabolites and their biotransformation routes for LDN-193189 are presented in Table 1. Microsomal fractions for all incubations were assessed using the positive control testosterone, which showed the expected metabolite formation.

### *In vitro* Metabolite Formation of Compounds **1**, **2**, and **3** in Human and Mouse Liver Subcellular Fractions

**FIGURE 5:**
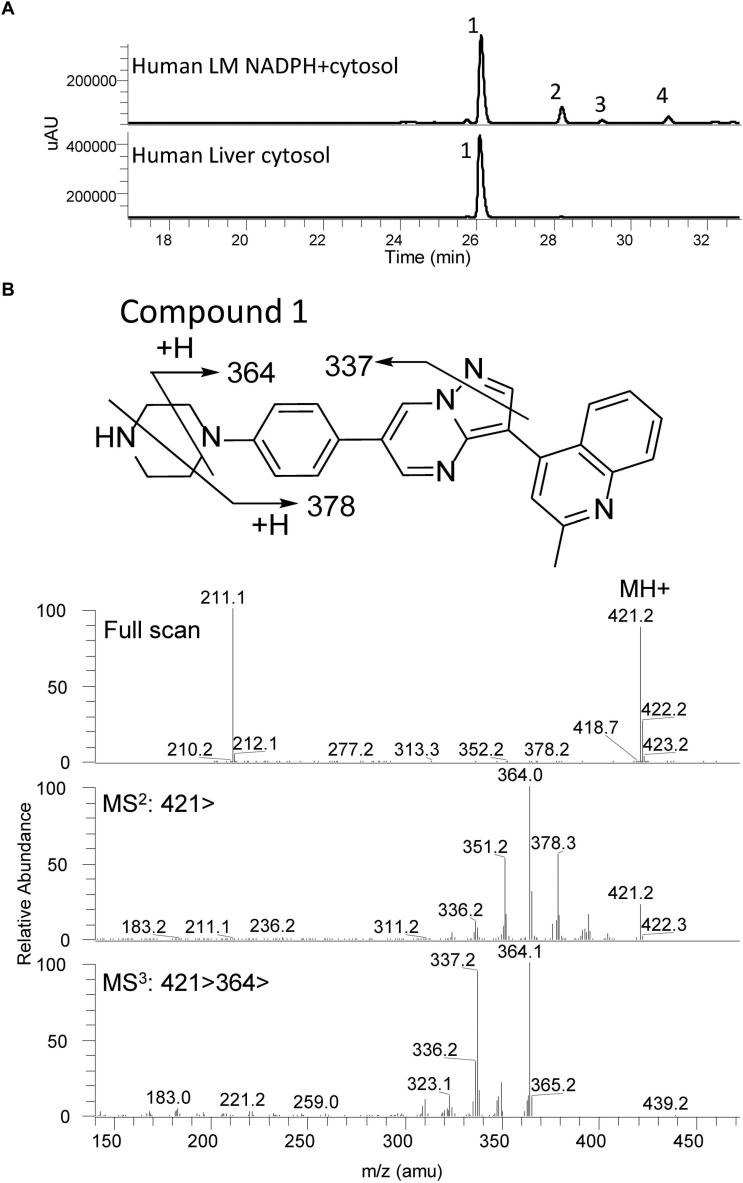
Metabolic profiles (LC/UV) of Compound **1** in human liver microsomes (LM) and cytosol. No AO-mediated metabolite was detected in the cytosol incubation. The main metabolic site was found to be at the piperazine moiety. Numbered peaks identified in the figure can be found in Table 2. **(A)** Metabolic profiles (LC/UV) of Compound **1** (λ = 370–380 nm for UV detection). **(B)** Fragment analysis of the parent drug.

The UV chromatograms of Compound **1** and the corresponding metabolic peaks generated by human liver microsomal and cytosolic fractions are presented in Figure 5A and Table 2. The compound appeared to be metabolized moderately in human liver microsomes. The major metabolic pathways were aliphatic hydroxylation of the piperazine ring to give M436a and M436b and further oxidation to M452. No metabolism was observed in the cytosol incubation, showing that methylation of the α-quinoline carbon blocked AO-mediated oxidation. No glucuronide conjugate was observed in UDPGA-fortified human liver microsomes. Also, no GSH or cyanide adduct was detected in trapping experiments, indicating that no reactive intermediate was formed.

**Table 2:** Summary of metabolites of Compound **1**.

Peak reference	Parent compound/Metabolites	RT (min)	MH^+^	Biotransformation pathways
1	Compound **1**	26.0	421	–
2	M436a	28.4	437	Oxidation
3	M452	29.5	453	2x Oxidation
4	M436b	31.2	437	Oxidation

*Following incubations in human liver microsomal and cytosolic fractions.*

Compound **1** fragment analysis is presented in Figure 5B. Fragment analysis of the metabolites can be found in Supplementary Table 2. No aniline derivatives were observed in human microsomal fractions. However, the piperazine moiety remains a sensitive site for bioactivation of the molecule and may have the possibility to generate aniline products *in vivo* since this moiety was not modified.

**FIGURE 6:**
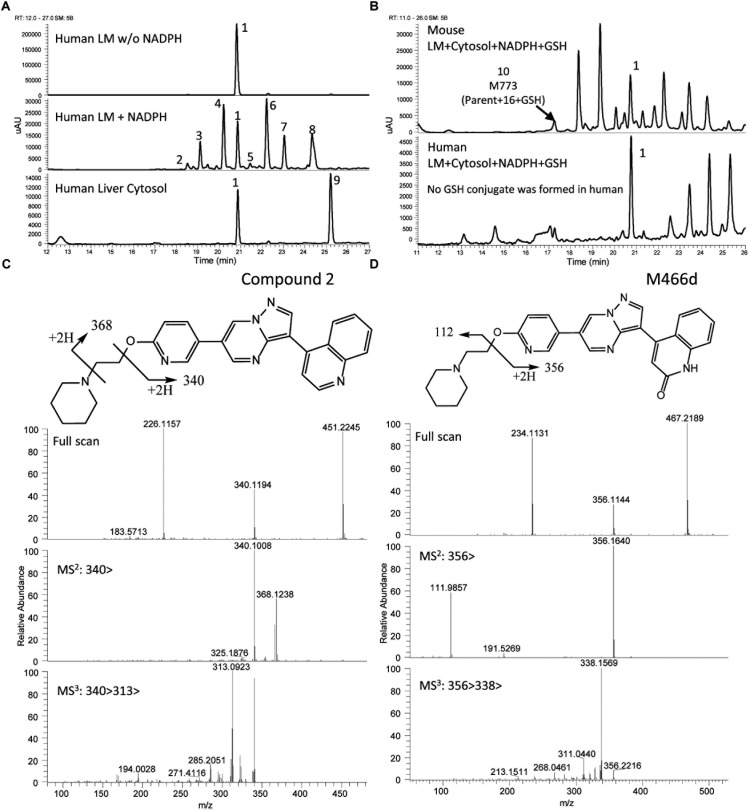
Metabolic profiles (LC/UV) and key metabolite formation of Compound **2**. AO mediated metabolism was observed (M466d, peak 9), and many oxidized and dealkylated metabolites were also detected. Numbered peak references in the figure can be found in Table 3. **(A)** Metabolic profiles (LC/UV) of Compound **2** in human liver microsomes (LM) and cytosol (LC/UV, λ = 370–380 nm). **(B)** LC/UV profiles of incubation containing GSH, the mouse sample showed a GSH adduct while the human sample did not. **(C)** Fragment analysis of parent Compound **2**. **(D)** Fragment analysis of main metabolite M466d, the AO-mediated product.

The UV chromatograms of Compound **2** and the corresponding metabolite peaks in human and mouse are presented in Figure 6 and Table 3. The metabolism of this molecule was mainly on the piperidine moiety, with the formation of several mono and multi-oxidized metabolites. An oxidative metabolite was observed in the cytosolic incubation (Figure 6A), suggesting that AO mediated metabolism occurred, most likely on the quinoline moiety to produce M466d (Figure 6D). A GSH adduct, M773, was observed only in mouse microsomal incubation in the presence of NADPH as shown in Figure 6B.

**Table 3:** Metabolite summary of Compound **2**.

Peak reference	Parent compound/Metabolites	RT (min)	MH^+^	Biotransformation pathways	Species
1	Compound **2**	20.6	451	–	H, M
2	M382	18.5	383	*N,N*-dealkylation	H, M
3	M466a	19.1	467	Oxidation	H, M
4	M482a	19.5	483	2x Oxidation	H, M
5	M482b	21.5	483	2x Oxidation	H, M
6	M383	22.2	384	*N,N*-dealkylation, Oxidative deamination	H, M
7	M466c	23.1	467	Oxidation	H, M
8	M397	24.3	398	N-dealkylation, Oxidation to carboxylic acid	H, M
9	M466d	25.2	467	Oxidation	H, M
10	M773	17.30	774	Oxidation, Glutathione adduct	M

*Following incubations in mouse and human liver microsomal and cytosolic fractions. M, Mouse; H, Human.*

Unlike LDN-193189, aniline or KCN adducts were not detected for Compound **2** as a result of the structure optimization which replaced the piperazine with piperidine. CYP mediated oxidation of the (piperidin-1-yl)ethoxy moiety occurred to produce metabolites M466a, M466b, M482a, M482c, M496, M480, and M446c. Oxidative deamination occurred to form M382, M383 and M397. For detailed LC/MS descriptions of the parent molecule and its proposed metabolite structures see Supplementary Table 3.

**FIGURE 7:**
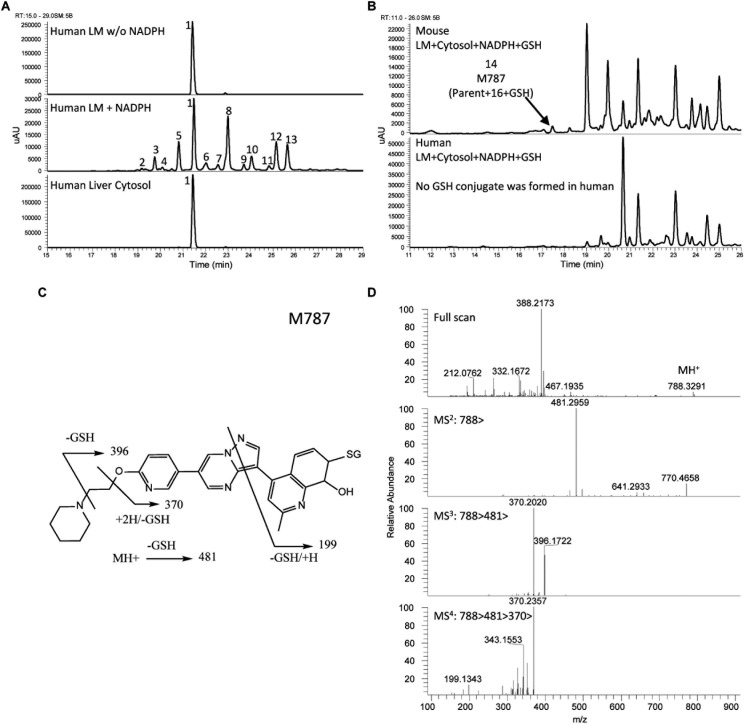
Metabolite profiles (LC/UV) of Compound **3** in human liver microsomes (LM) and cytosol and fragment analysis of parent compound. Number references in the figure can be found in Table 4. **(A)** Metabolite profiles (LC/UV) of Compound **3** with λ = 370–380 nm. **(B)** LC/UV profiles of incubation containing GSH, mouse sample presents a GSH adduct, human sample does not. **(C)** Fragmentation scheme of M787. **(D)** MS fragmentation spectra of M787.

The UV chromatograms of Compound **3** and the corresponding metabolite peaks are presented in Figure 7A and Table 4. No metabolite formation was observed in the cytosol incubation, and the main site of metabolism in the microsome incubation was on the piperidine moiety forming many mono and multi-oxidized metabolites. A GSH adduct M787 was observed only in mouse microsomal incubation in the presence of NADPH as shown in Figure 7B. No cyanide adduct or glucuronide conjugate was observed.

**Table 4:** Summary of metabolites of Compound **3**.

Peak reference	Parent compound/Metabolites	RT (min)	MH^+^	Biotransformation pathways	Species
1	Compound **3**	21.3	465	–	H, M
2	M396	19.2	397	*N,N*-dealkylation	H, M
3	M480a	19.7	481	Oxidation	H, M
4	M480b	20.1	481	Oxidation	H, M
5	M496a	20.8	497	2x Oxidation	H, M
6	M413a	21.9	414	*N,N*-dealkylation, oxidative deamination, Oxidation	H, M
7	M496b	22.5	497	2x Oxidation	H, M
8	M397	23.0	398	*N,N*-dealkylation, Oxidative deamination	H, M
9	M510a	23.7	511	3x Oxidation, Desaturation	H, M
10	M494	24.1	495	2x Oxidation, Desaturation	H, M
11	M510b	24.8	511	3x Oxidation, Desaturation	H, M
12	M411	25.2	412	*N*-dealkylation, Oxidation to carboxylic acid	H, M
13	M413b	25.7	414	*N,N*-dealkylation, Oxidative deamination, Oxidation	H, M
14	M787	17.47	788	Oxidation, Glutathione adduct	M

*Following incubations in human and mouse liver microsomal and cytosolic fractions. M, Mouse; H, Human.*

**FIGURE 8:**
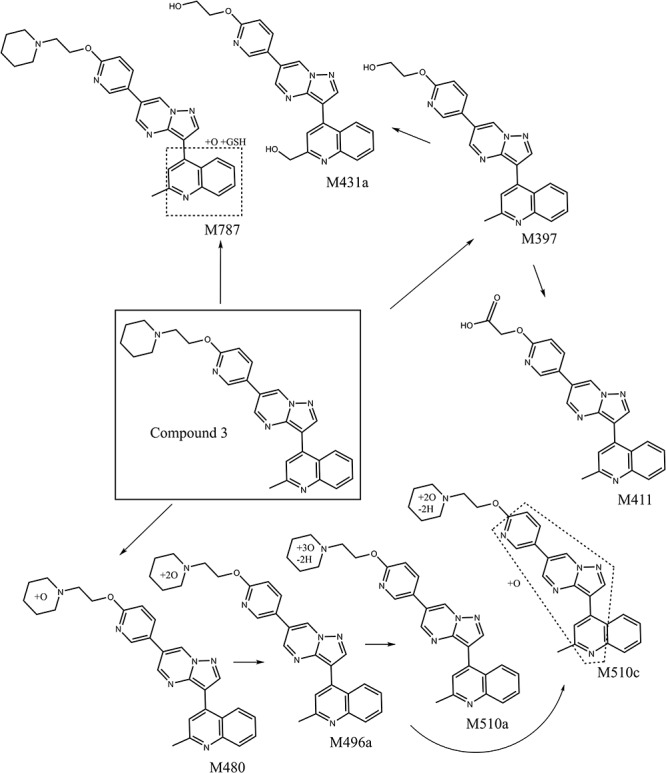
Proposed metabolic pathways of optimized backup Compound **3** after incubation with liver microsomal and cytosolic fractions from mice and humans. No aniline or aldehyde oxidase mediated metabolites were observed.

Compound **3** MetID revealed several oxidation/hydroxylation sites, mainly on the piperidine ring. Metabolites M480a, M480b, M494 M496a, M496b, and M510a are examples. Neither aniline formation nor cytosolic metabolism were observed. Piperidine ring opening generated the metabolites M396, M397, and M411. Acetaminophen, a positive control for GSH trapping experiments, also showed GSH trapped products in both mouse and human incubations. The metabolic pathway of Compound **3** is shown in Figure 8. Compound **3**’s metabolite M787 fragment analysis is shown in Figure 7C,D. Additional LC/MS information on each structure can be found in Supplementary Table 4.

### *In vitro* ADME and Activity Properties

To assess whether the modifications made to LDN-193189 were not causing loss of activity or affecting critical ADME properties, physicochemical characteristics, cell-based activity and *in vitro* ADME properties were measured and are shown in Table 5.

**Table 5:** Physicochemical characteristics, *in vitro* activity and ADME properties for LDN-193189 compared to backup compounds.

	Physicochemical characteristics			Activity			*In vitro* ADME		
Compound ID	clogP	pKa	TPSA^∗^	BMP6 (mediated by ALK2) IC_50_ (nM)	BMP4 (mediated by ALK3) IC_50_ (nM)	TGFβ (mediated by ALK5) IC_50_ (nM)	Microsomal Stability t_1/2_ (min)	Permeability (10^−6^ cm/s)	Solubility (μg/mL)
LDN-193189	3.7	8.6	55.6	157	585	14670	>30	320	1.1
Compound **1**	4.2	8.6	55.6	120	727	987	>30	437	1.0
Compound **2**	4.7	9.0	65.1	227	3268	27420	22	7	<1.0
Compound **3**	5.3	9.0	65.1	466	4336	2664	>30	1069	36.2

*Physicochemical characteristics were calculated using ChemDraw Professional (Ver.16.0). ^∗^Topological Polar Surface Area.*

As shown in Table 5, the methylation of the quinoline moiety only slightly affected the calculated physicochemical characteristics of the compounds. From LDN-193189 to Compound **1**, the methylation increased clogP from 3.7 to 4.2, and a similar effect occurred between Compounds **2** and **3**, with clogP increasing from 4.7 to 5.3. Methylation did not affect the pKa and the Topological Polar Surface Area (TPSA).

Compared to LDN-193189, Compound **3** was only threefold less potent against ALK2 mediated signaling. Selectivity in the BMP4 assay improved, while selectivity in the TGFβ assay was decreased. Its solubility was considerably increased despite the increase in clogP; there was also a significant improvement in the permeability of Compound **3** compared to the other analogs. Compound **2** with the same (pyridin-2-yloxy)ethyl)piperidine group as Compound **3**, was less metabolically stable than it, and the methylation of the quinoline group in Compound **3** appears to account for its increased metabolic stability. Overall, the *in vitro* results of Compound **3** showed improved *in vitro* ADME properties while retaining acceptable activity.

## Discussion

The *in vitro* metabolism study results show that LDN-193189 is extensively metabolized by the cytosolic enzyme AO in the majority of the species studied, with a minor contribution from NADPH-dependent microsomal metabolism. The major metabolite formed in the presence of cytosol was identified as NIH-Q55 (M422c). A variety of metabolites were identified in the microsomal fraction incubations, mainly resulting from metabolism at the piperazine moiety. Metabolites M431 and M445 were only observable in KCN trapping experiments. Therefore these metabolites’ precursors are likely to be hard electrophiles. The detection of an aniline metabolite M337 raised drug safety concerns as the association of aniline with genotoxicity has been widely reported (Bentzien et al., 2010). No unique human metabolite was formed *in vitro*. To address the metabolic liabilities of the aniline and reactive metabolite formation, as well as the AO-mediated metabolism as the major metabolic pathway for LDN-193189, additional structure optimization has been undertaken.

The AO-mediated formation of M422c is probably the reason for the relative short half-life of LDN-193189 *in vivo* (Yu et al., 2008a). This was further confirmed by measuring M422c *in vivo* concentrations in mouse PK studies (data not shown). Moreover, the metabolite profile obtained from human liver microsomes combined with cytosol showed a significant dependence on AO as the CYP450 mediated metabolism was relatively minor (see Figure 2A). Notwithstanding the short half-life, AO mediated metabolism may represent a concern regarding a drug’s eventual success as observed by Zetterberg and co-authors in the study of VX-509 (decernotinib) (Zetterberg et al., 2016). In the VX-509 case, preclinical studies did not consider AO-mediated metabolism. When the molecule reached human clinical trials, unforeseen drug-drug interactions were observed and later discovered to be caused by a metabolite exclusively formed by AO. This resulted in serious restrictions on the use of the new drug. Also, AO susceptibility is a liability owing to high inter-subject variability since the level of activity of this enzyme may vary up to 90-fold from patient to patient (Kitamura et al., 1999; Hutzler et al., 2014).

Another concern about the high contribution of AO to LDN-193189 metabolism is the selection of preclinical animal species for drug safety evaluations. AO is differently expressed across mammalian species (Terao et al., 2006; Choughule et al., 2013; Dalvie et al., 2013). For example, dogs are known to be deficient or have low AO activity (Terao et al., 2006; Jensen et al., 2017). In this study, the extent of metabolic conversion to M422c appeared to be in the order of monkey > rabbit > human > mouse > rat with no conversion observed in dog liver preparations. Our findings corroborate the activity of AO since the metabolic profiles of LDN-193189 across the species were consistent with the natural abundance of AO in the different species.

Because of these liabilities, new analogs were designed to avoid the AO metabolic pathway. Various strategies can be employed to address LDN-193189 AO liability, such as the replacement of the quinoline moiety (Jiang et al., 2018). Here, the strategy was to block the α-carbon of the quinoline moiety with a methyl group. Therefore, Compound **1** was synthesized and the *in vitro* MetID study confirmed that this compound was not susceptible to AO activity in the presence of cytosol (Figure 5). The modification caused an increased clogP from 3.7 in LDN-193189 to 4.2 in Compound **1**, without compromising ALK2 IC_50_ and *in vitro* ADME properties.

LDN-193189 was effectively metabolized by microsomal enzymes such as NADPH dependent CYP450s. The rank ordering of the metabolic conversion in the presence of liver microsomes (no cytosol added) was dog > rabbit > mouse > human ∼ monkey > rat. Following incubations with liver microsomes, LDN-193189 was mainly metabolized on the piperazine moiety, producing various piperazine ring-cleaved derivatives including M337, M380, M381, M395, and M438. A piperazine ring-oxidized derivative (lactam) M420 was also demonstrated in all species. Metabolites M422a and M422b appear to be N-oxide/hydroxylamine analogs of LDN-193189 based on the mass spectral data obtained with H/D exchange, as shown in Figure 3D. Metabolites M422a and M422b showed the same number of exchangeable protons as the parent compound. In contrast, metabolite M422c, which is proposed to be formed by oxidation at a carbon, leads to an additional exchangeable proton due to the hydroxyl group.

Bioactivation of LDN-193189 to form reactive intermediates was demonstrated by the presence of cyanide adducts in liver microsomal extracts from all species. Cyanide conjugate M431 and a further oxidized derivative, M445, were observed in all species when KCN was added to the incubation mixtures. Based on the mass spectral data, the bioactivation was localized to the piperazine moiety. It is proposed that an iminium derivative of the piperazine moiety was produced, which subsequently was trapped by cyanide. There is ample precedence in the literature for the formation of iminium species from alicyclic amines such as nicotine (Murphy, 1973; Gorrod and Aislaitner, 1994). Nicotine was used as the positive control during our trapping experiments and it produced the expected cyanide derivatives. The reactive MetID strategy has been employed with gefitinib and a proposed reactive iminium species possibly contributes to the adverse effects of the drug (Liu et al., 2015). Trapping studies conducted with GSH showed that there were no “soft electrophiles” produced from LDN-193189 in the presence of liver microsomes from any species.

A piperazine is a structural alert in a molecule owing to its propensity to be bioactivated to generate iminium species. Further, the piperazine in LDN-193189 also went through N-dealkylation to form the aniline derivative M337. Aniline toxicity is widely reported in the literature and it is a structural alert (Kalgutkar et al., 2008; Di Girolamo et al., 2009; Mašiè, 2011; Kalgutkar, 2015). Aniline compounds are bioactivated by CYP enzymes to generate reactive oxygen species (ROS) that may cause cell damage (Wang et al., 2016). In general, aniline derivatives are oxidized to N-hydroxylamine, which can be further oxidized to the nitrosobenzene metabolite, a soft electrophile that may react with proteins. Also, phase II metabolism of N-hydroxylamine could lead to additional reactive species with the potential to cause cell damage. N-hydroxylamine is known to be mutagenic (Harrison and Jollow, 1986; Bentzien et al., 2010; Kalgutkar, 2015).

To avoid the bioactivation and further dealkylation of the piperazinyl ring to form the aniline, an alkylether linked substituent was used at the same position as the piperazine ring. Also, replacing the phenyl with an electron-deficient pyridinyl ring would further reduce the potential for aromatic ring oxidation. Moreover, the addition of more metabolic soft spots would shift the metabolic pathways and reduce the potential for generation of reactive species (Mašiè, 2011). To avoid aniline and reactive species formation, LDN-193189 was further optimized by incorporating the aforementioned features, culminating in the discovery of Compound **2**. In Compound **2**, the phenylpiperazine moiety of LDN-193189 was replaced by a 4-(2-(pyridin-2-yloxy)ethyl)piperidine group. The MetID of this compound showed that that moiety was subject to extensive NADPH-dependent metabolism but did not form reactive metabolites. Altogether, this molecule cannot generate an aniline metabolite and it was much less likely to form iminium reactive species compared to LDN-193189, as shown in Figure 6. However, the compound had reduced metabolic stability against rat liver microsomes. Finally, as a proof of concept, we synthesized Compound **3**, which combined the strategies used in Compounds **1** and **2** to address both AO liability and aniline formation. The result, Compound **3**, successfully avoided the metabolism concerns while retaining comparable, selective activity for BMP6 and improved ADME properties as compared to LDN-193189.

The MetID technique, as illustrated in this study, pinpointed liabilities in the LDN-193189 molecule and guided further lead optimization required to obtain a compound devoid of AO susceptibility and toxic metabolite formation. MetID is growing as an important tool in drug discovery and lead optimization (Zak et al., 2015; Obach et al., 2016). Metabolism studies should not ignore aldehyde oxidase enzyme activity as an important metabolic pathway that may hinder the success of novel compounds (Garattini and Terao, 2011; Zetterberg et al., 2016; Jensen et al., 2017). Also, due to high species differences in AO activities, *in vitro* studies with S9, microsomal and/or cytosolic fractions must consider the use of material from different species. Such practice may also guide the selection of a preclinical animal model (Dalvie et al., 2013).

Currently, several therapeutics for FOP are being pursued. ALK2 inhibitors such as K02288, DMH1 and other derivatives of LDN-193189 (LDN-214117, LDN-212854) were recently evaluated (Mohedas et al., 2013, 2014); inhibitors of ACVR1 gene expression are being tested (Cappato et al., 2016); palovarotene, a retinoic acid receptor γ (RARγ) agonist, was efficacious in animal models (Chakkalakal et al., 2016) and is currently in phase III Clinical Trials (Clementia Pharmaceuticals, NCT02279095); and an activin A antibody is currently in Phase II Clinical Trials (Regeneron Pharmaceuticals, NCT033112634).

We aim to contribute to the discovery of FOP therapeutics with an emphasis on the application of MetID during the lead optimization in discovery phase. Our work on the structure optimization of LDN-193189 demonstrates the importance of MetID for guiding chemical structure design of a desirable drug candidate.

## Author Contributions

EP, XX, EK, JW, AM, and PS participated in research design. EP, JW, and AM conducted the experiments. WH, JJ, and AL provided the compound synthesis. EP, XX, JW, AM, and JJ performed the data analysis. EP, JW, AL, AM, WH, RP, PY, JM, PS, and XX wrote or contributed to the writing of the manuscript.

## Conflict of Interest Statement

JW was employed by company Janssen Research and Development. AM was employed by company Frontage Laboratories. The remaining authors declare that the research was conducted in the absence of any commercial or financial relationships that could be construed as a potential conflict of interest.

## Abbreviations

ACVR1: activin A receptor type 1
ALK: anaplastic lymphoma kinase
AO: aldehyde oxidase
BMP: bone morphogenetic protein
CYP: Cytochrome P-450
FMO: flavin-containing monooxygenase
FOP: fibrodysplasia ossificans progressiva
HLM: human liver microsomes
MoCo: molybdenum cofactor
NIH-Q55: synthetic reference standard of M422c metabolite
RARγ: retinoic acid receptor γ
XO: xanthine oxidase.
