# Supplementary material for: Application of in vitro Drug Metabolism Studies in Chemical Structure Optimization for the Treatment of Fibrodysplasia Ossificans Progressiva (FOP)
Source: Front Pharmacol. 2019 Apr 24;10:234. doi: 10.3389/fphar.2019.00234 (PMC6491728; doi:10.3389/fphar.2019.00234)
Supplement: Supplementary file 1 [file Table_1.DOCX]

Supplementary Material

**Application of In Vitro Drug Metabolism Studies in Chemical Structure Optimization for the Treatment of Fibrodysplasia Ossificans Pogressiva (FOP)**

Elias C Padilha, Jianyao Wang, Ed Kerns, Arthur Lee, Wenwei Huang, Jian-kang Jiang, John McKew, Abdul Mutlib, Rosangela G Peccinini, Paul Yu, Philip Sanderson and Xin Xu*

*** Correspondence:**Xin Xu, PhD
[xin.xu3@nih.gov](mailto:xin.xu3@nih.gov)

**Supplementary Table 1** – Product ions of LDN-193189 and metabolites

| **Parent Compound/**  **Metabolites** | **Retention**  **Time (min)** | **Proposed Metabolite**  **Identification** | **Product Ions** |
| --- | --- | --- | --- |
| **LDN-193189** | 30.20 |  | 407.2, 380.1, 364.1, 350.1, 337.2, 322.1, 310.1, 169.0 |
| M337 | 27.45 |  | 338.2, 311.1, 264.1, 294.1, 169.0 |
| M438 | 28.10 |  | 439.2, 421.1, 381.2, 363.1, 350.1, 337.1 |
| M380 | 29.93 |  | 381.2, 364.1, 350.1, 337.1, 322.1, 323.1, 296.1, 168.1 |
| M422a | 33.38 |  | 423.2, 335.3, 212.2, 405.1, 364.1, 350.1, 323.1, 169.1 |
| M381 | 32.65 |  | 382.2, 364.1, 350.1, 337.1, 310.1, 284.1, 168.1 |

**Supplementary Table 1** – Product ions of LDN-193189 and metabolites (Cont.)

| **Parent Compound/**  **Metabolites** | **Retention**  **time (min)** | **Proposed Metabolite**  **Identification** | **Product ions** |
| --- | --- | --- | --- |
| M420 | 33.55 |  | 421.2, 350.1, 323.1, 295.2, 211.2 |
| M431 | 34.09 |  | 432.1, 405.1, 377.0, 350.1, 323.1 |
| M422b | 37.03 |  | 423.2, 406.1, 364.1, 212.3 |
| M422c | 39.78 |  | 423.1, 212.2, 380.0, 362.0, 337.1, 261.2, 237.1, 320.1 |
| M395 | 39.18 |  | 396.2, 352.1, 338.1, 311.1, 284.1, 194.1 |
| M445 | 41.02 |  | 446.2, 419.1, 391.1, 350.1, 323.0 |

**Supplementary Table 2** – Product ions of Compound 1 and metabolites

| **Parent Compound/**  **Metabolites** | **Retention**  **Time (min)** | **Proposed Metabolite**  **Identification** | **Product Ions** |
| --- | --- | --- | --- |
| **Compound 1** | 26.04 |  | 421.2, 378.3, 364.0, 351.2, 337.2 |
| M436a | 28.36 |  | 437.2, 396.2, 378.2, 364.2, 337.2 |
| M452 | 29.49 |  | 435.2, 364.2, 337.2 |
| M436b | 17.47 |  | 437.2, 420.2, 378.1, 363.1 |

## Supplementary Table 3 – Product ions of Compound 2 and metabolites

| **Parent Compound/**  **Metabolites** | | **Retention**  **Time (min)** | | | **Proposed Metabolite**  **Identification** | | **Product Ions** | | |  |
| --- | --- | --- | --- | --- | --- | --- | --- | --- | --- | --- |
| **Compound 2** | | 20.64 | |  | | 451.2, 368.1, 340.1, 226.1(2+), 285.2, 313.1 | | |  |  |
| M382 | | 18.49 | |  | | 383.2, 340.1, 366.3, 313.1, 285.2 | | |  |  |
| M466a | | 19.13 | |  | | 467.2, 449.3, 366.2, 340.1, 234.1(2+), 313.1 285.2 | | |  |  |
| M466b | | 19.50 | |  | | 467.2, 340.1, 234.1(2+), 313.1, 285.2, 128.1 | | |  |  |
| M466c | | 23.05 | |  | | 467.2, 356.1, 339.1, 234.1(2+), 112.1 | | |  |  |
| M482a | | 20.23 | |  | | 483.2, 340.1, 313.1, 285.1, 242.1, 144.0 | | |  |  |
| M482b | | 21.48 | |  | | 483.2, 356.1, 242.1(2+), 339.2, 128.0 | | |  |  |
| M482c | | 23.68 | |  | | 483.2, 340.1, 313.1, 285.2, 144.0 | | |  |  |
| M383 | 22.22 | |  | | | | | 348.1, 366.1, 340.2, 313.1, 285.2 | | |
| M480 | 23.38 | |  | | | | | 481.2, 340.1, 313.1, 285.2 | | |
| M496 | 24.14 | |  | | | | | 497.2, 340.2, 313.2, 285.1, 158.0 | | |
| M397 | 24.35 | |  | | | | | 398.1, 340.1, 313.2, 285.2 | | |
|  |  | |  | | | | |  | | |
|  |  | |  | | | | |  | | |

## Supplementary Table 3 – Product ions of Compound 2 and metabolites (Cont.)

| **Parent Compound/**  **Metabolites** | **Retention**  **Time (min)** | **Proposed Metabolite**  **Identification** | **Product Ions** |
| --- | --- | --- | --- |
| M466d | 25.19 |  | 467.2, 356.2, 338.1, 234.1(2+), 112.0 |
| M773 | 17.30 |  | 774.3, 467.3, 384.1, 356.2, 185.2 |

**Supplementary Table 4** – Product ions of Compound 3 and metabolites

| **Parent Compound/**  **Metabolites** | **Retention**  **Time (min)** | **Proposed Metabolite**  **Identification** | **Product Ions** |
| --- | --- | --- | --- |
| **Compound 3** | 21.25 |  | 465.2, 354.1, 233.1(2+), 190.5(2+), 327.2, 112.0 |
| M396 | 19.21 |  | 397.2, 354.1, M380.2, 327.2 |
| M480a | 19.74 |  | 481.2, 463.3, 354.1, 241.1(2+), 380.2, 354.2, 327.2 |
| M480b | 20.08 |  | 481.2, 380.2, 354.1, 241.1(2+), 327.2, |
| M496a | 20.78 |  | 497.2, 354.1, 249.1(2+), 327.2 |
| M496b | 22.48 |  | 497.2, 479.4, 396.1, 379.2, 370.2, 354.2 |
| M496c | 24.32 |  | 497.2, 354.2, 327.1, 144.0 |
| M413a | 21.97 |  | 414.2, 396.3, 352.1 |
| M413b | 25.70 |  | 414.2, 397.2, 353.2 |
| M397 | 22.97 |  | 398.2, 380.3, 354.3, 327.1 |
| M510a | 23.68 |  | 511.2, 354.3, 327.1 |

**Supplementary Table 4** – Product ions of Compound 3 and metabolites (Cont.)

| **Parent Compound/**  **Metabolites** | **Retention**  **Time (min)** | **Proposed Metabolite**  **Identification** | **Product Ions** |
| --- | --- | --- | --- |
| M510b | 24.82 |  | 511.2, 354.1, 327.1, 158.1 |
| M510c | 26.67 |  | 511.2, 370.2, 353.26 |
| M494 | 24.09 |  | 495.2, 354.1, 327.2 |
| M411 | 25.18 |  | 412.1, 354.2, 327.2 |
| M787 | 17.43 |  | 788.3, 481.3, 396.2, 388.2, 370.0, 343.15, 199.1 |
